# A Rare Case of Ipsilateral Radial Head, Neck, and Shaft Fracture With Intact Ulna in a Young Adult

**DOI:** 10.7759/cureus.93446

**Published:** 2025-09-28

**Authors:** Satyanarayana Pidikiti, Kavyansh Bhan, Asif Parkar

**Affiliations:** 1 Trauma and Orthopaedics, Barking, Havering and Redbridge University Hospitals NHS Trust, London, GBR; 2 Trauma and Orthopaedics, University Hospitals Dorset NHS Foundation Trust, Bournemouth, GBR

**Keywords:** elbow trauma, forearm injury, proximal radius, radial head fracture, radial neck fracture, radius shaft fracture

## Abstract

Fractures involving multiple levels of the radius are rare and often associated with ulnar involvement or distal radius injury. We report a previously undocumented injury pattern: a comminuted ipsilateral fracture of the radial head, neck, and shaft, with an intact ulna, in a 28-year-old male following road traffic trauma. The classification system of the Arbeitsgemeinschaft für Osteosynthesefragen (AO), also known as the Working Group for Osteosynthesis Questions, does not include this type of fracture configuration. The patient underwent open reduction and internal fixation using a headless screw construct for the radial head and a mini fragment locking plate spanning from the shaft to the radial head. Despite the rare pattern of injury and complexity involved, the patient achieved satisfactory early functional outcomes. This case highlights the importance of recognising rare radial fracture patterns and employing tailored fixation strategies to restore function, anatomical alignment, and stability.

## Introduction

Radial head and neck fractures are among the most common elbow injuries in adults, typically caused by a fall onto an outstretched arm with valgus force [1]. Shaft radius fractures are more frequently associated with direct trauma or rotational forces [2]. Simultaneous multi-level injuries of the radius, particularly involving the head, neck, and shaft, are exceedingly rare and often involve the ulna or distal radius [3,4]. Injury patterns involving the entire radial column, while sparing the ulna, are scarcely reported. Some variants, like 'bipolar' injuries (radial head and distal radius fractures), have been described [5,6]. Other reports include trifocal radius fractures involving the radial head, shaft, and distal radius [7,8]. However, to our knowledge, there are no previous reports of an ipsilateral fracture involving the radial head, radial neck, and shaft, with an intact ulna. We present such a case and discuss its unique surgical considerations and management outcomes.

## Case presentation

**Figure 1:**
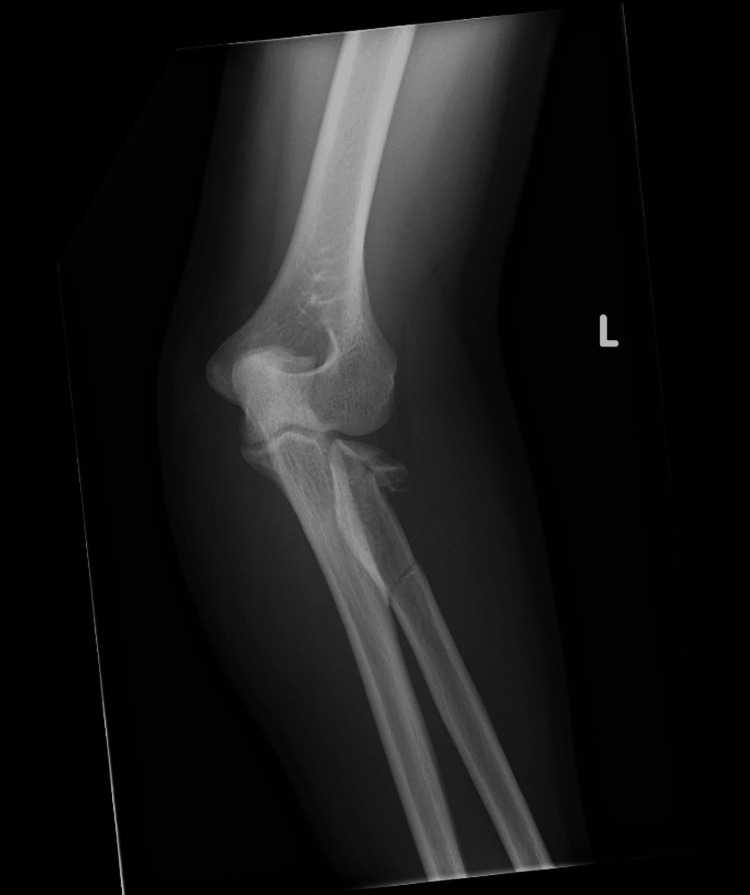
Anteroposterior view of the elbow injury radiograph

**Figure 2:**
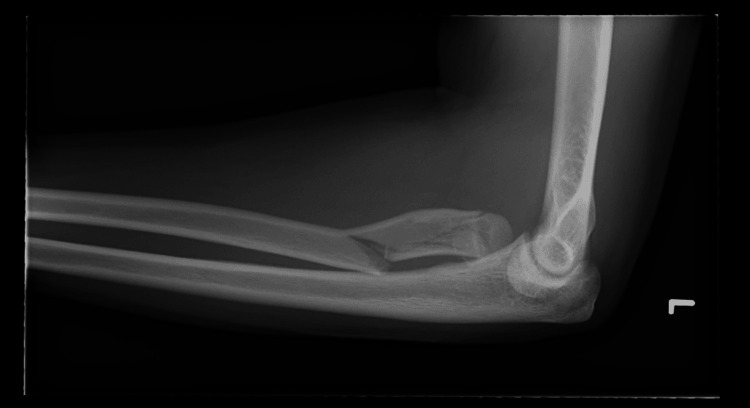
Lateral view of the elbow injury radiograph

**Figure 3:**
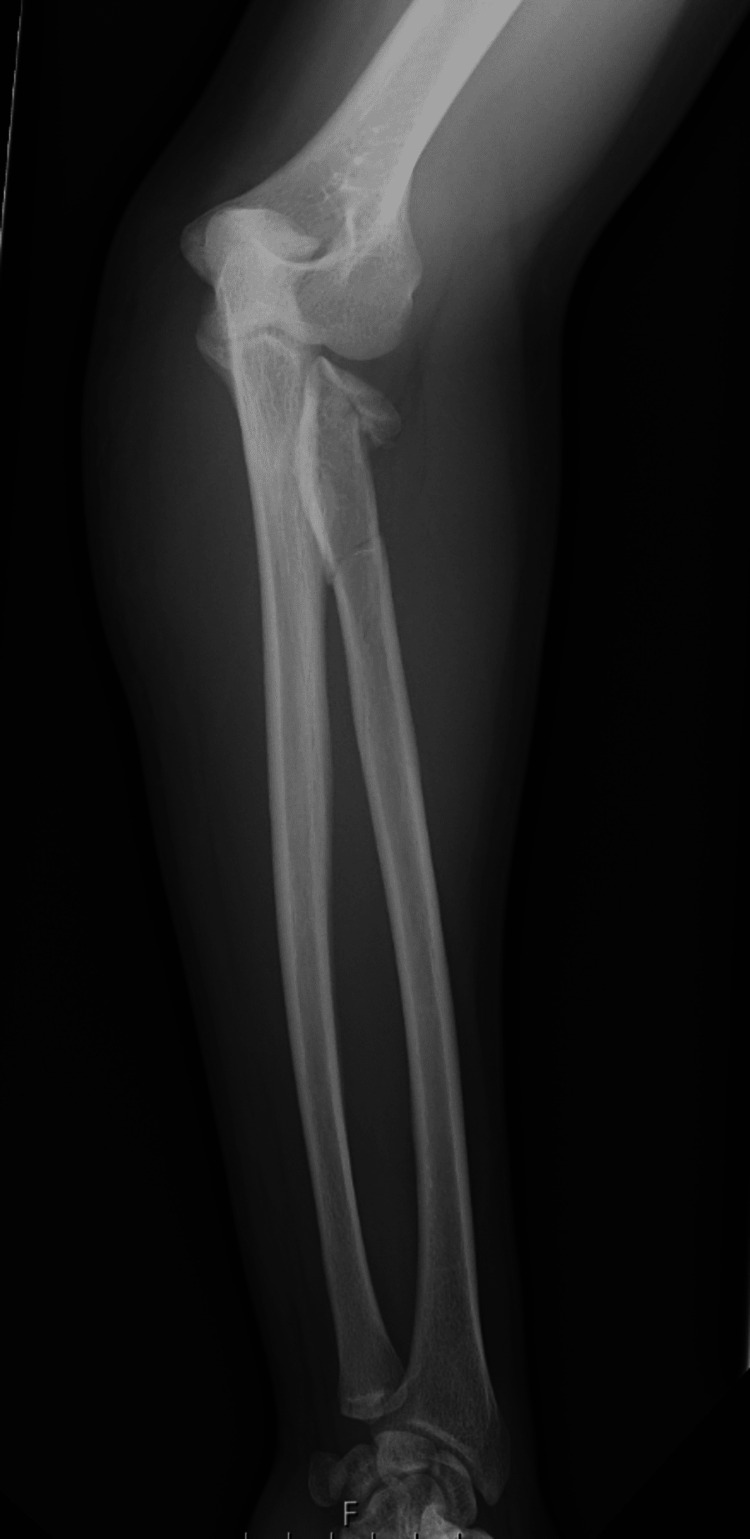
Preoperative radiograph confirming no distal radius/ulna fractures

**Figure 4:**
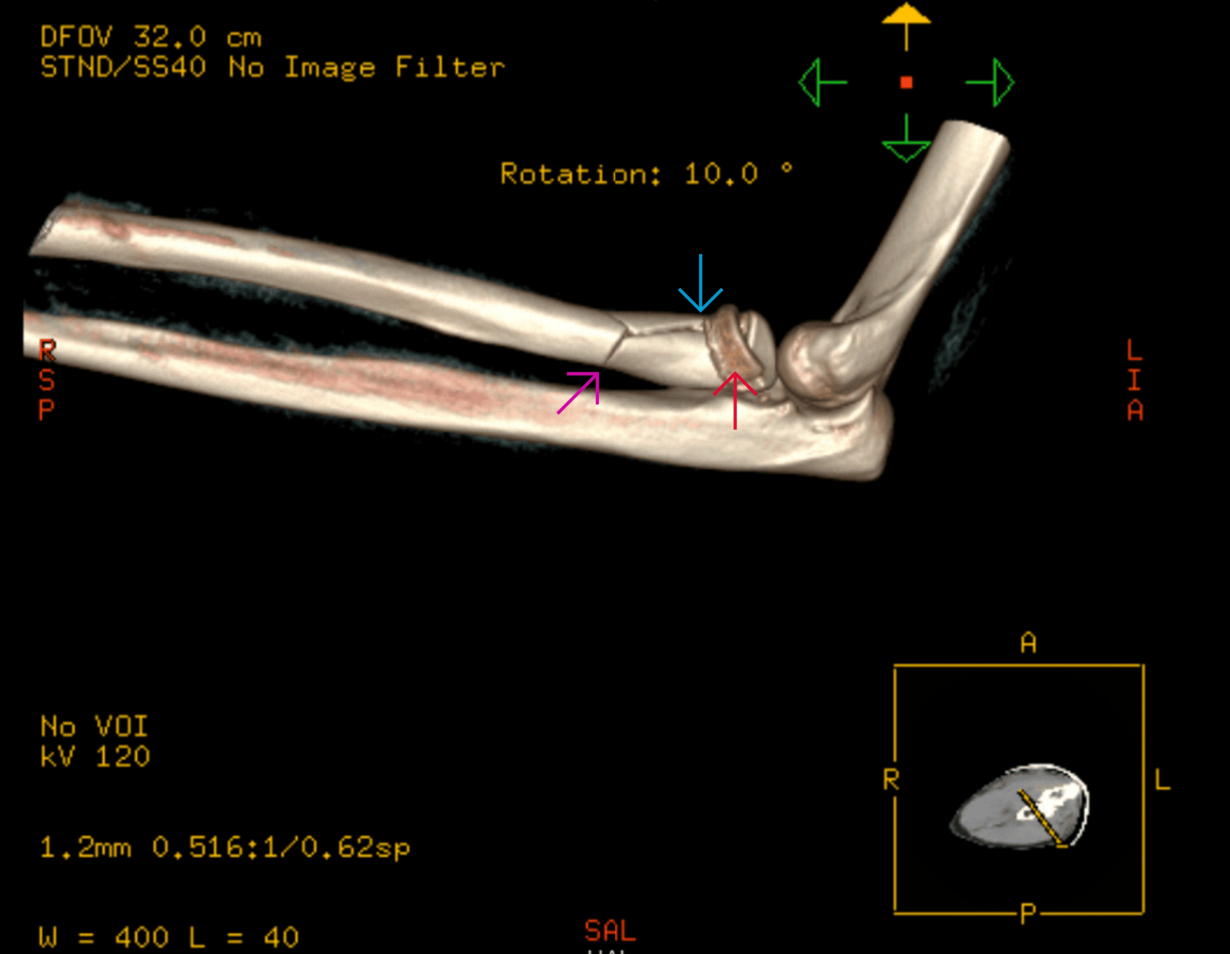
CT scan image showing the radial head (red arrow), neck (blue arrow), and shaft (pink arrow) fracture with intact ulna

A 28-year-old right-hand-dominant male presented after a high-speed road traffic accident with pain, swelling, and restricted motion in his left forearm and elbow. The skin was intact, and the neurovascular status was normal. This was an isolated injury. After a thorough Advanced Trauma Life Support (ATLS) assessment, the patient had plain radiographs and a subsequent CT scan, which revealed a comminuted fracture of the radial head (transverse fracture with bone loss) along with a radial neck fracture with impaction into the shaft and a spiral and longitudinal fracture of the radial shaft (Figures 1-4). No ulna fracture was demonstrated in the radiographs and CT scan (Figures 3-4). Following initial stabilisation in an above-elbow backslab, the patient underwent open reduction and internal fixation (ORIF) under general anaesthesia on the following day.

Surgical management

**Figure 5:**
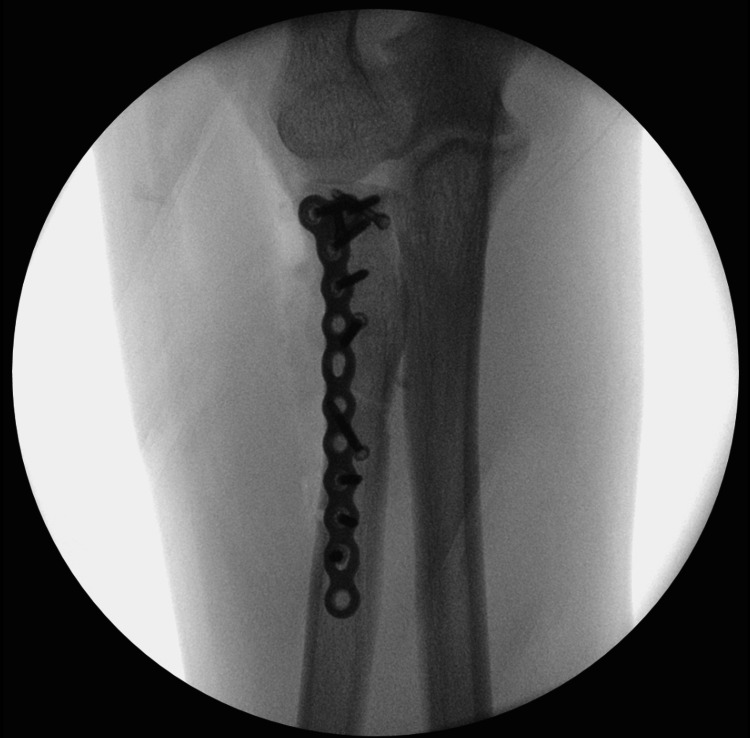
Anteroposterior view of intraoperative fluoroscopy demonstrating fixation

**Figure 6:**
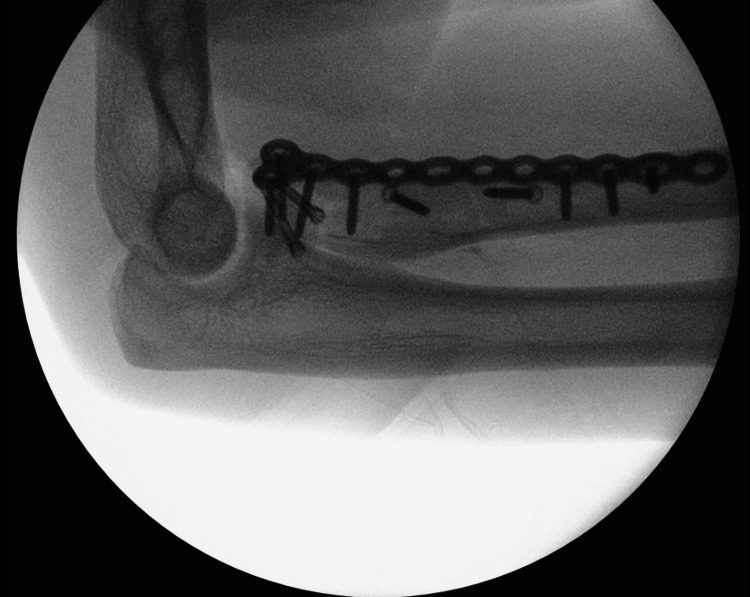
Lateral view of intraoperative fluoroscopy demonstrating fixation

The patient was operated on in a supine position with the elbow extended on an arm board. A Kaplan approach was used to access the proximal radius [9]. The interval between the extensor carpi radialis brevis and the extensor digitorum communis was developed. Comminution and bone loss at both the radial head and shaft were noted. Also observed was a transverse radial head fracture and impaction of the radial neck into the shaft. The same incision was extended distally to expose the radial shaft. The posterior interosseous nerve (PIN) was identified and protected using a vascular loop. A spiral and longitudinal fracture of the midshaft with a butterfly fragment was noted. The radial head was reduced and stabilized using 2.5 mm headless compression screws. The radial neck and shaft were reconstructed using a variable-angle mini fragment locking T-plate. Additional 2.7 mm cortical lag screws were used to improve shaft compression (Figures 5-6). The radial head was noted to be stable in full supination and pronation with minimal capsular release. Intraoperative fluoroscopy showed good alignment. Intraoperative testing demonstrated a near full range of motion, comparable to the contralateral limb. The elbow joint was found to be stable to both varus and valgus testing, with no mechanical blocks noted on supination or pronation.

**Figure 7:**
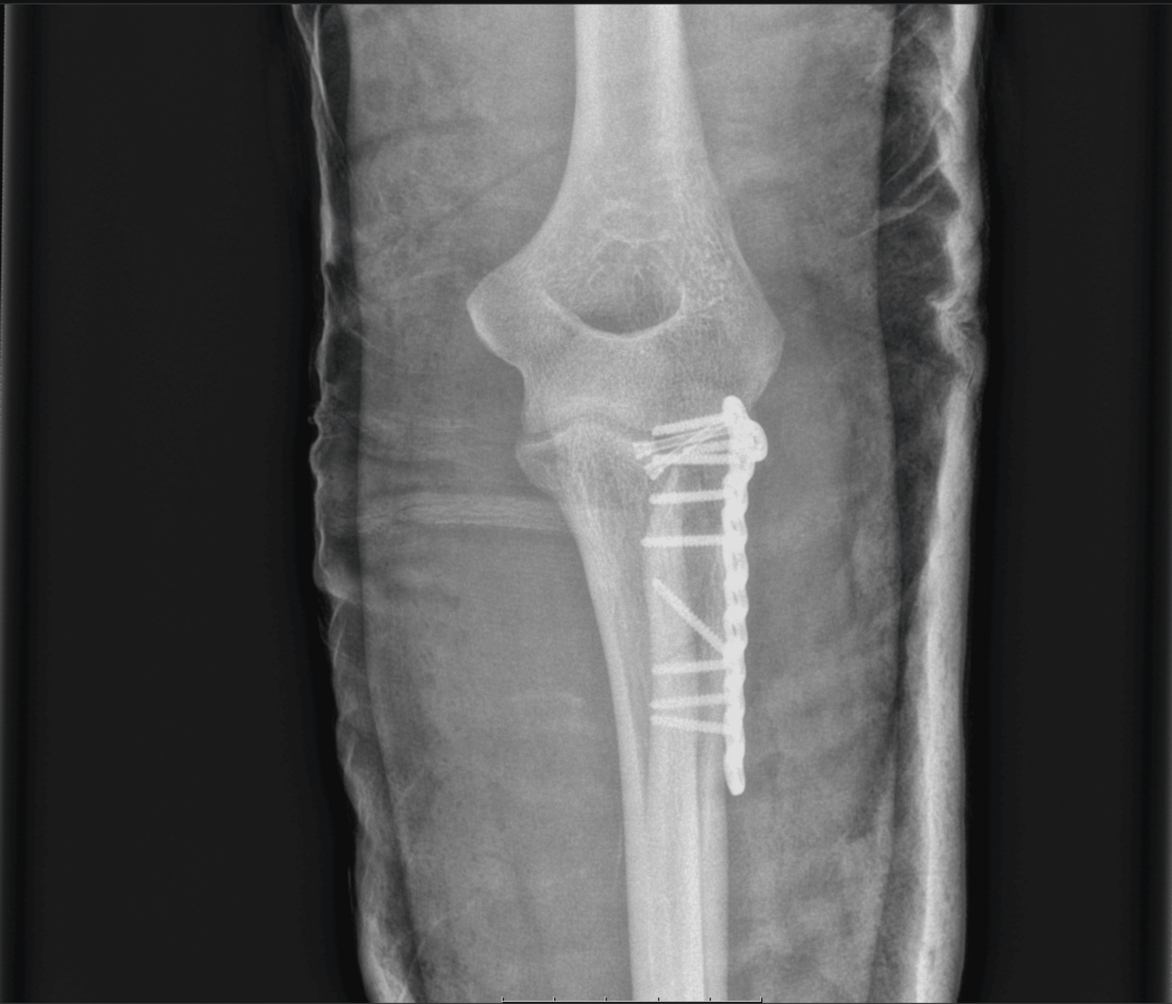
Anteroposterior view of postoperative radiograph at two weeks

**Figure 8:**
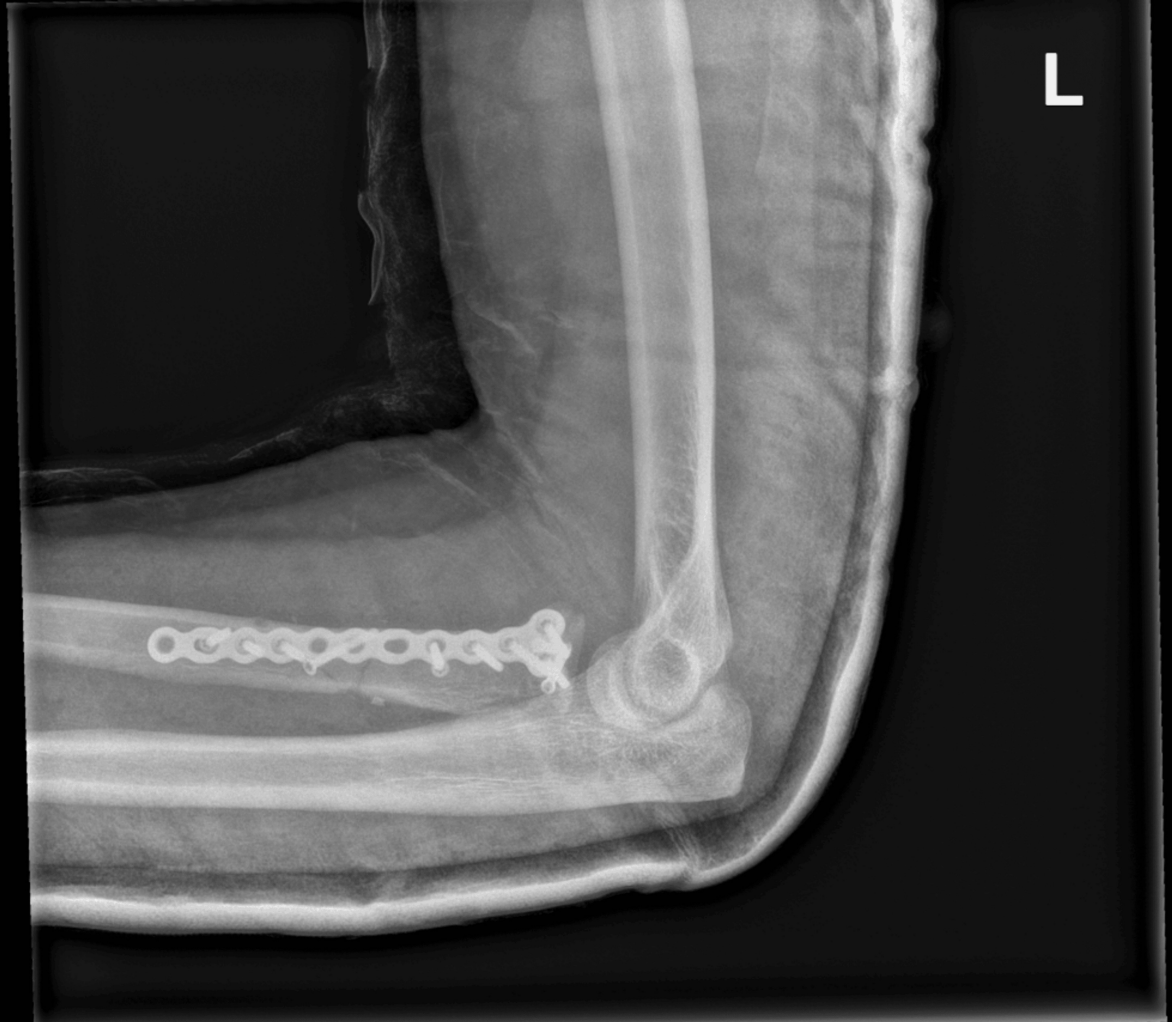
Lateral view of postoperative radiograph at two weeks

Postoperatively, the patient's forearm was held in an elevated position in a sling with regular monitoring for compartment syndrome until discharge later in the evening. Postoperative management included short-term immobilisation in an above-elbow backslab for two weeks, followed by structured physiotherapy (Figures 7-8). There was no neurovascular deficit. At three months, the patient achieved 0-130° elbow flexion, 70° pronation, and 75° supination. The patient completed the Disabilities of the Arm, Shoulder, and Hand (DASH) questionnaire [10] and scored 10 on a scale measured from zero to 100; a higher score indicates greater disability. Radiographs confirmed maintenance of metalwork and alignment at follow-up.

## Discussion

This case demonstrates a previously unreported trifocal radial injury sparing the ulna. The unique pattern, complexity of bone loss, and limited literature underscore important challenges in upper limb trauma surgery. A high-energy axial load with valgus force likely caused a comminuted fracture of the radial head; the impaction of the neck into the shaft with the rotational force propagating distally produced a spiral shaft fracture. The isolated radial injury, without ulnar disruption, suggests the ulna acted as a stabilizer and absorbed none of the deforming force, which is unusual for such high-energy mechanisms [11]. Previous literature has described either radial head and distal radius (bipolar) injuries [5,6], or radial head dislocation with shaft fracture [4,12]. There have been papers discussing ipsilateral trifocal radius fractures, but these often involve the distal radius or ulna [7,8]. None have described comminuted radial head, radial neck, and shaft fractures with an intact ulna.

In this case, the goal of operative fixation was to restore radial column alignment for forearm rotation, achieve articular congruity of the radial head, and provide rigid shaft stabilization. The use of headless screws allowed joint-preserving fixation, as per Hotchkiss and Ring’s recommendations [13,14]. A long T-plate bridging the radial shaft to the head provided additional load-sharing and rotational control. The stability of the radial head on testing likely suggests minimal annular ligament and interosseous membrane injury. Soft tissue healing over time and early rehabilitation restored function without any episode of dislocation, which is dissimilar to the outcomes reported in complex Monteggia variants [15,16].

## Conclusions

This case presents an exceptionally rare trifocal radial fracture involving the radial head, neck, and shaft, occurring in isolation without ulnar involvement. This is an injury pattern that has not been previously documented in the literature to the best of our knowledge. Recognising this unique injury is crucial, as it spans multiple zones along the radial column and demands a comprehensive surgical approach. 

This case highlights the importance of individualised fixation strategies based on fracture morphology and the effectiveness of headless screws and mini fragment systems in managing complex fractures in anatomically challenging regions such as the proximal radius. It contributes to the limited literature on complex forearm injuries and serves as a reference for orthopaedic surgeons managing similar high-energy, multi-segment radial fractures.
